# Association between GIS-Based Exposure to Urban Air Pollution during Pregnancy and Birth Weight in the INMA Sabadell Cohort

**DOI:** 10.1289/ehp.0800256

**Published:** 2009-04-13

**Authors:** Inmaculada Aguilera, Mònica Guxens, Raquel Garcia-Esteban, Teresa Corbella, Mark J. Nieuwenhuijsen, Carles M. Foradada, Jordi Sunyer

**Affiliations:** 1 Centre for Research in Environmental Epidemiology, Barcelona, Spain; 2 Municipal Institute of Medical Research (IMIM-Hospital del Mar), Barcelona, Spain; 3 Centro de Investigación Biomédica en Red de Epidemiología y Salud Pública, Barcelona, Spain; 4 Pompeu Fabra University, Barcelona, Spain; 5 Servei de Salut, Ajuntament de Sabadell, Sabadell, Spain; 6 Servei de Ginecologia i Obstetrícia, Hospital de Sabadell, Sabadell, Spain

**Keywords:** air pollution, aromatic hydrocarbons, birth weight, cohort study, exposure assessment, geographic information system, INMA study, nitrogen dioxide, pregnancy

## Abstract

**Background:**

There is growing evidence that traffic-related air pollution reduces birth weight. Improving exposure assessment is a key issue to advance in this research area.

**Objective:**

We investigated the effect of prenatal exposure to traffic-related air pollution via geographic information system (GIS) models on birth weight in 570 newborns from the INMA (Environment and Childhood) Sabadell cohort.

**Methods:**

We estimated pregnancy and trimester-specific exposures to nitrogen dioxide and aromatic hydrocarbons [benzene, toluene, ethylbenzene, *m*/*p*-xylene, and *o*-xylene (BTEX)] by using temporally adjusted land-use regression (LUR) models. We built models for NO_2_ and BTEX using four and three 1-week measurement campaigns, respectively, at 57 locations. We assessed the relationship between prenatal air pollution exposure and birth weight with linear regression models. We performed sensitivity analyses considering time spent at home and time spent in nonresidential outdoor environments during pregnancy.

**Results:**

In the overall cohort, neither NO_2_ nor BTEX exposure was significantly associated with birth weight in any of the exposure periods. When considering only women who spent < 2 hr/day in nonresidential outdoor environments, the estimated reductions in birth weight associated with an interquartile range increase in BTEX exposure levels were 77 g [95% confidence interval (CI), 7–146 g] and 102 g (95% CI, 28–176 g) for exposures during the whole pregnancy and the second trimester, respectively. The effects of NO_2_ exposure were less clear in this subset.

**Conclusions:**

The association of BTEX with reduced birth weight underscores the negative role of vehicle exhaust pollutants in reproductive health. Time–activity patterns during pregnancy complement GIS-based models in exposure assessment.

Fetal growth is an important indicator of the health of newborns and infants that may influence the health status in the adulthood (Sinclair et al. 2007). In recent years, a growing body of research has associated prenatal exposure to air pollution with adverse pregnancy outcomes, including intrauterine growth restriction (IUGR), low birth weight (LBW), preterm birth (PTB), and intrauterine mortality. Detailed reviews of these studies have concluded that the strength of the evidence differs among air pollutants, birth outcomes, and exposure periods, although differences in study design, exposure assessment, and definition of outcomes make comparability of results difficult (Glinianaia et al. 2004; Lacasaña et al. 2005; Šrám et al. 2005; Wang and Pinkerton 2007).

To advance in this emerging and fast-growing field, some key methodologic issues have been highlighted (Gilliland et al. 2005; Ritz and Wilhelm 2008; Slama et al. 2008a). Because most studies have linked birth outcomes and covariates from birth certificate records with routinely measured air pollutants, one priority is to develop prospective cohort studies that are able to obtain high-quality individual data on outcomes, covariates, and exposure estimates. Because pregnancy is a well-defined and relatively narrow period of exposure, identification of windows of greater susceptibility to air pollution is also a key issue, but is difficult because of the lack of biological knowledge and the correlations among trimester- or month-specific exposures. Furthermore, exposure assessment can be improved by using approaches based on geographic information systems (GIS) that take into account small-area variations in vehicle exhaust pollutants, such as land-use regression (LUR). Because LUR models have mainly been used for estimating annual average exposures, being able to accurately incorporate temporal variability in the LUR models is key for studies of birth outcomes, where shorter-term exposures are of interest. Improving exposure assessment also requires consideration of women’s residential mobility (Fell et al. 2004) and time–activity patterns during pregnancy (Nethery et al. 2009).

In this study we assessed the relationship between GIS-based exposure to traffic-related air pollution during pregnancy and birth weight in an urban cohort from the Spanish INMA (Environment and Childhood) Study. We also examined the influence of time– activity patterns during pregnancy in the association between air pollution and birth weight.

## Methods

### Cohort

The study area is Sabadell, a city of nearly 200,000 inhabitants situated in the metropolitan area of Barcelona, Spain. Women who visited the public health center of Sabadell in the 12th week of pregnancy and fulfilled the inclusion criteria were eligible to participate in the study (Ribas-Fitó et al. 2006). Main exclusion criteria were being < 16 years of age, nonsingleton pregnancy, not planning to deliver at the Hospital of Sabadell, and having followed an assisted reproduction program. Women were interviewed in the 12th and 32nd weeks of pregnancy and answered several questionnaires on sociodemographic characteristics, health status, use of drugs, occupational data, environmental exposures, time–activity patterns, and a food-frequency questionnaire. The protocol of the INMA study, including a detailed description of data collection and assessment of determinants and outcomes, has been published elsewhere (Ribas-Fitó et al. 2006). The study was approved by the Ethical Committees of the Municipal Institute of Medical Research and the Hospital of Sabadell, and all subjects gave written informed consent before participating.

A total of 657 women were enrolled in the study between June 2004 and July 2006. This sample was representative of the target population in terms of women’s attendance at prenatal care in the public health system (used by 85% of the pregnant women in Sabadell), but the educational level of our sample was higher than the target population average. From the initial sample, we followed 619 (94%) women until the child’s birth. We excluded 44 children from the analysis because their mothers did not live in Sabadell during pregnancy but in nearby cities covered by the health service of the municipal hospital. We also excluded three children with no recorded birth weight and two with gestational duration of 28 and 32 weeks, respectively, because of missing data in the covariates obtained in the 32nd week interview. Finally, 570 (87%) children were included in the analysis.

### Air pollution exposure model

We used LUR modeling in the study area to estimate individual exposure to nitrogen dioxide and BTEX (benzene, toluene, ethylbenzene, *m*/*p*-xylene, and *o*-xylene) as markers of motor vehicle exhaust pollution. A complete description of the methodology on exposure modeling has been reported previously (Aguilera et al. 2008). Briefly, we measured NO_2_ and BTEX with passive samplers in four and three sampling campaigns of 1 week, respectively, between April 2005 and March 2006, conducted simultaneously at 57 sampling sites (29 urban background and 28 traffic sites) representing the gradient of exposure in the study population. For each pollutant, we calculated average concentrations of all the sampling campaigns, assuming that they are representative of annual mean levels of NO_2_ and BTEX (Lebret et al. 2000), and fitted linear regression models using five groups of geographic data (land coverage, topography, population density, roads, and distance to local sources of pollution) as predictor variables. Geographic variables were stored and derived in ArcGIS, version 9.1 (ESRI, Redlands, CA, USA). The final model for NO_2_ (*R*^2^ = 0.75) included altitude, road type (major, secondary, or minor road), and a land cover factor within a 500-m buffer as predictor variables. Geographic variables included in the final BTEX model (*R*^2^ = 0.74) were altitude and three source-proximity variables (distance to nearest major road, secondary road, and parking lot). We used two cross-validation procedures to evaluate the precision of the regression models.

We then applied models to predict outdoor air pollution levels at the cohort addresses. For women who changed their home address during pregnancy (*n* = 25, 4%), exposure was calculated using the estimated concentrations at both the old and new home address, weighted by the percentage of the pregnancy period spent in each of them.

We adjusted models for temporal variations to calculate term-specific individual exposures, as it has been done in other studies relying on LUR exposure models (Brauer et al. 2008; Slama et al. 2007). To obtain an average exposure for the whole pregnancy period and for each trimester, we used temporal variations of air pollution measured in the only fixed monitoring station operating in Sabadell. The station is located on a traffic island in the middle of a main road, in a relatively open area with unobstructed air flow. Daily measurements of air pollution conducted simultaneously in this traffic site and in an urban background location during 1 month showed similar temporal variations in NO_2_ levels between the two sites, with a correlation coefficient of 0.96 (Rivas-Lara 2008). We averaged daily means of NO_2_ measured at the fixed station over the pregnancy period for each woman. The resulting value was divided by the average NO_2_ concentration corresponding to the whole sampling period (from April 2005 to March 2006) and multiplied by the predicted value obtained in the LUR model. We applied the same procedure to estimate trimester-specific exposures for each woman. We defined the first trimester of pregnancy as weeks 1–13, the second trimester as weeks 14–26, and the third trimester as the period from week 27 until birth.

Regarding BTEX, the fixed monitoring station measures daily mean levels of benzene and toluene, but the high percentage of missing data (65% of the sampling period) did not allow us to “seasonalize” the BTEX model with them. Because NO_2_ showed higher correlation with benzene and toluene in the fixed monitoring station than with other traffic-related pollutants such as carbon monoxide or particulate matter (PM), and given the high correlation between NO_2_ and BTEX levels measured with the passive samplers (*r* = 0.80 for the whole sampling period), we used NO_2_ daily levels to make the temporal adjustment, assuming that the temporal variations in both pollutants were similar.

### Birth weight and gestational age

Birth weight was recorded by specially trained midwives at delivery. We calculated gestational age from the date of the last menstrual period (LMP) reported at recruitment and confirmed using estimates based on ultrasound examination in the 12th week of gestation. When the difference between the LMP reported at recruitment and estimated from the ultrasound was ≥ 7 days (*n* = 91; 16%), we estimated LMP using a quadratic regression formula defined by Westerway et al. (2000).

### Statistical analysis

We examined the association between birth weight and prenatal exposure to NO_2_ and BTEX by simple and multiple linear regression models. Other reproductive outcomes such as LBW or small for gestational age were not considered for analysis because of the relatively low sample size. Given the high correlation among the five BTEX compounds (*r* > 0.75) and because the relative fetal toxicity of each of them is not well known (Agency for Toxic Substances and Disease Registry 2004), we used the LUR estimate of the sum of the five compounds to assess the relationship between BTEX exposure and birth weight.

We chose covariates included in the analysis based on previous knowledge on their influence on birth weight. We collected some through questionnaire in the two interviews carried out during pregnancy for each woman: Maternal age, maternal education, maternal ethnicity, parity, maternal height and prepregnancy weight, and paternal height and weight were obtained in the 12th-week interview; tobacco use and passive smoking information were collected in the 32nd-week interview. Birth date and child sex were collected from the child’s neonatal anthropometry record filled in by the midwives. We calculated season of conception using date of LMP. Because season of birth is influenced by the duration of pregnancy, we used season of conception in the analysis rather than season of birth.

With linear regression models, we estimated the change in birth weight for an interquartile range (IQR) increase in NO_2_ and BTEX exposure (micrograms per cubic meter), for each trimester and for the entire pregnancy. We retained as adjustment factors only those covariates that modified the association between air pollution and birth weight by > 10%. Because fetal weight gain per week is not constant throughout pregnancy, we examined the association between birth weight and gestational age by using fractional polynomial models to identify the best-fit transformation of gestational age and allow polynomial terms for gestational age in the linear regression models (Blair et al. 2005).

We performed a sensitivity analysis considering time–activity patterns during pregnancy. In the 32nd-week interview, women answered the following question for a typical weekday and for a typical weekend: “Since you have gotten pregnant, how much time have you typically spent daily in these environments?” The answer options were (a) home indoors, (b) work indoors, (c) in other people’s houses, (d) in other indoor environments, (e) home outdoors, (f) work outdoors, (g) in other outdoor environments, and (h) in means of transportation. The question was designed to obtain a 24-hr sum. We weighted the data to account for weekdays (5 of 7) and weekends (2 of 7) and then calculated time spent at home (answers a + e) and time spent in non-residential outdoor environments (answers f + g). We used the median (rounded to the nearest whole number) as a cutoff value to restrict our analysis to two subsets: *a*) women who spent more time at home and *b*) women who spent less time in nonresidential outdoor environments. Because we based LUR estimates on the women’s residential addresses, we assumed that these two subsets suffered less from exposure misclassification and that misclassification was nondifferential.

We performed statistical analyses using Stata 8.2 (StataCorp., College Station, TX, USA).

## Results

Mean birth weight of included births was 3,247 g (10th, 50th, and 90th percentiles: 2,721, 3,288, and 3,760 g), and mean maternal age was 31.4 years (minimum and maximum, 18.2 and 43 years, respectively). Table 1 shows other characteristics of the study population and mean birth weight for each categorized variable. Birth weight was associated (*p* < 0.10) with child’s sex, season of conception, parity, tobacco smoke, passive smoking, maternal ethnicity, gestational age, maternal height and prepregnancy weight, and paternal height and weight.

We examined whether air pollution exposure was associated with maternal education as a surrogate of socioeconomic status (SES). We found a small but statistically significant association between LUR estimates of BTEX levels and maternal education (*p* = 0.02). Predicted annual mean levels of BTEX were 17.6, 16.0, and 16.1 μg/m^3^ for women with a university degree, secondary education, and primary education, respectively. The corresponding NO_2_ values for the three categories were 37.4, 35.7, and 35.7 μg/m^3^ (*p* = 0.14).

Tables 2 and 3 provide the distribution of 9-month and trimester-specific exposures to NO_2_ and BTEX and the correlation coefficients among them, respectively. We found only slight differences between mean exposure levels by trimester and 9-month exposures, although the range of exposure was wider for the three trimester exposures than for the whole pregnancy period. According to these estimates, 14% of the women had an average NO_2_ exposure > 40 μg/m^3^ for the entire pregnancy period, which is the European Union limit value to come into force in 2010 (European Commission 1999). Correlation coefficients among the three trimesters ranged from 0.45 to 0.50 for NO_2_ and from 0.72 to 0.74 for BTEX, reflecting small seasonal variation in exposure.

Table 4 shows time–activity patterns reported in the 32nd-week interview and referring to the entire pregnancy. Differences between weekdays and weekends were statistically significant for all the activities. During weekdays, women who did not work during pregnancy or worked only during part of it (*n* = 350) spent more time at home and in nonresidential outdoor environments, and less time in means of transportation, compared with women who worked during the entire pregnancy (*n* = 210) (Mann–Whitney test, *p* < 0.05). We found no differences in total time spent in indoor environments between the two groups.

Table 5 presents the effect of air pollution exposure during pregnancy and during each trimester on birth weight. Neither NO_2_ nor BTEX exposure was significantly associated with the outcome in any of the exposure periods. Associations for BTEX were more pronounced in the subset of women who spent ≥ 15 hr/day at home (*n* = 276), but they were also not statistically significant. However, when considering only women who spent < 2 hr/day in nonresidential outdoor environments (*n* = 259), BTEX exposure both during the whole pregnancy period and the second trimester showed a statistically significant negative effect on birth weight. Estimated reductions in birth weight for an IQR increase of BTEX exposure were 76.6 g and 101.9 g during pregnancy and the second trimester, respectively. The negative effect of NO_2_ exposure variables in this subset of women was less clear but showed some stronger effects during the second trimester of pregnancy (*p* = 0.09).

Because the three trimester exposures of both pollutants (particularly BTEX) were correlated, we also adjusted models for trimester-specific exposures (Table 5). Associations found for BTEX and NO_2_ exposures in the second trimester were more pronounced in the whole cohort and in the two subsets, but only statistically significant among women who spent < 2 hr/day in nonresidential outdoor environments. Variance inflation factor values ranged from 1.76 to 1.96 for NO_2_ and from 2.53 to 2.89 for BTEX, indicating acceptable levels of collinearity in the multi-trimester models.

## Discussion

We found an effect of exposure to BTEX, and to a lesser extent NO_2_, during the second trimester of pregnancy on birth weight among a subset of women who spent < 2 hr/day in outdoor environments during pregnancy, after controlling for exposure to the same pollutant during the other two trimesters. Exposure to BTEX during the whole pregnancy period was also significantly associated with birth weight for the same subset. The magnitude of the association was higher for BTEX in all the exposure periods. Overall, exposure during the second trimester appeared to be the most harmful, and the association became larger after adjusting for trimester-specific exposures.

Identifying critical exposure windows is a research need but a difficult task because of differences in mixture of pollutants across space and time, as well as possible different effects of specific pollutants during specific exposure periods (Slama et al. 2008a). In addition, there is currently a lack of toxicologic information to help guide selection of relevant exposure periods for most fetal growth end points (Ritz and Wilhelm 2008). To our knowledge, this is the first study assessing the relationship between prenatal exposure to ambient BTEX and birth weight, so we cannot compare our results with those of other studies. Regarding NO_2_, the evidence of a susceptible window of exposure is unclear. Some studies found an adverse effect of NO_2_ on birth weight in the second trimester of pregnancy (Lee et al. 2003; Mannes et al. 2005), whereas others identified first trimester of exposure to NO_2_ as the only period influencing fetal growth, measured as continuous birth weight, LBW, or IUGR (Bell et al. 2007; Ha et al. 2001; Salam et al. 2005). Two studies found an association between NO_2_ and birth weight for the whole pregnancy but did not identify any specific harmful exposure period (Brauer et al. 2008; Liu et al. 2007). Finally, other studies did not observe any significant association between NO_2_ and fetal growth (Gouveia et al. 2004; Hansen et al. 2007; Liu et al. 2003; Slama et al. 2007). However, between-study comparisons are limited by differences in study design, exposure assessment, and different outcome definitions (IUGR or birth weight treated as continuous or dichotomous variable).

We found reductions in birth weight with increases in BTEX concentrations only among women who spent < 2 hr/day in nonresidential outdoor locations. This could potentially be due to less exposure misclassification (assumed nondifferential) in residence-based LUR estimates for this subset. Although representing a small portion of total daily activity, time spent outdoors can signify direct exposure to traffic-related pollutants. Thus, women who spent a considerable amount of time (≥ 2 hr/day) in nonresidential outdoor environments could have been exposed to a high variability of traffic-related NO_2_ and BTEX levels, very different than those reflected by the LUR estimates based on the residential address. This hypothesis is supported by results obtained for a subset of 53 women of this cohort in their third trimester of pregnancy, selected to represent the geographic distribution of the cohort addresses, and for which personal levels of NO_2_ were measured with passive samplers during 48 hr. In this subset, women who spent ≥ 2 hr/day in nonresidential outdoor environments (reported for the 48-hr measurement period) showed higher personal levels of NO_2_ (β = 14.4 μg/m^3^; 95% confidence interval, 4.6–24.3 μg/m^3^), compared with the reference group (< 2 hr/day) (Valero N, Aguilera I, Llop S, Esplugues A, de Nazelle A, Ballester F, et al., unpublished observations). A study conducted in Athens also found that time spent outdoors in the city center was a major contributor to personal exposure to toluene and xylenes (Alexopoulos et al. 2006). Nethery et al. (2008) found a better correlation (*r* = 0.72) between 48-hr personal exposure to nitric oxide and LUR-estimates based on home address in a subset of pregnant women who spent > 65% of sampling time at home, compared with those who spent ≤ 65% (*r* = 0.31). Although not statistically significant, effect estimates for BTEX in our cohort were also more pronounced among women who spent more time at home, compared with the whole cohort. Overall, results reinforce the need of considering time–activity patterns during pregnancy to better characterize the exposure (Ritz and Wilhelm 2008).

The inclusion of LUR estimates based on work addresses also could improve exposure assessment among employed women (Nethery et al. 2008). In our study, we were unable to account for work-based LUR estimates for the whole subset of employed women because approximately 25% of them worked outside the area covered by our LUR models and 16% reported imprecise work addresses that we were unable to geocode. We did not conduct a sensitivity analysis by working status because 37% (*n* = 160) of the 437 women who were employed at the beginning of the study changed their working status during the 12th- and 32nd-week interviews, making the trimester-specific classification of working status in this subset prone to error, particularly for the second trimester of pregnancy. Instead, we investigated differences in time–activity patterns by working status during the whole pregnancy and found that women who worked during the entire pregnancy spent less time in nonresidential outdoor environments. This suggests that this time–activity variable, although reported mainly as a walking activity, is not an indicator of commuting but of a wider variety of transit activities.

Several studies have reported seasonal patterns both in air pollution levels and in birth weight (Hazenkamp-von Arx et al. 2004; Murray et al. 2000). In Sabadell, daily mean levels of NO_2_, benzene, and toluene (measured at the fixed monitoring station) were higher in winter and lower in summer during the study period, probably due to seasonal differences in meteorologic conditions and traffic intensity. We also have found a seasonal pattern in birth weight, with lowest birth weights seen in infants conceived in winter. This effect is larger than that observed in other studies (Jedrychowski et al. 2004; Slama et al. 2007; Wilhelm and Ritz 2005) and independent from the air pollution effects (*p* for interaction > 0.10), suggesting that seasonal effects on birth weight could be related to seasonal factors other than air pollution, such as ambient temperature and sunlight (Murray et al. 2000; Tustin et al. 2004).

LUR estimates of NO_2_ and BTEX levels were higher among women with higher educational level, compared with women with secondary or primary education. This could be explained by the higher percentage of women with university degree living in the city center, which is one of the districts with higher air pollution levels because of its higher road density and economic activity (Aguilera et al. 2008). O’Neill et al. (2003) found that people with lower SES tend to live in areas with higher levels of air pollution in North America. This evidence is more limited in Europe, with some studies suggesting that the inverse association may occur (Forastiere et al. 2007; Hoek et al. 2002). Differences between European and North American cities in their structure and social class distribution could explain these discrepancies. Within Europe, southern European cities have a more dense structure of roads and buildings, and thus higher traffic emissions, particularly in central districts (Muñoz 2003). Although preliminary, our results support the hypothesis that higher SES can be associated with living in more polluted areas in southern European cities.

One of the strengths of our study is estimation of individual exposure to traffic-related air pollutants based on temporally adjusted LUR models applied to geocoded home addresses, whereas most studies assess exposure by using routinely measured air pollution levels and community-level residence (census data or postal codes). To date, only two studies have also applied temporally adjusted LUR models in Munich, Germany, and Vancouver, Canada (Brauer et al. 2008; Slama et al. 2007), although they did not take into account time–activity patterns as potential factors affecting exposure misclassification. In addition, we accounted for residential mobility during pregnancy when assigning exposures, and we were able to control for a considerable number of potential confounders not always available in studies relying on data from birth certificates, such as quantitative measures of maternal smoking, passive smoking, or maternal and paternal weight and height.

Because we used ultrasound measurements to correct reported LMP dates that differed in ≥ 7 days from the ultrasound-based estimates, corrected gestational age could be biased if air pollution exposure shows an early effect on fetal growth (Slama et al. 2008b). However, analysis of gestational age (both corrected and noncorrected) by exposure categories during the first trimester indicated that the correction of gestational age was not biased by air pollution effects.

A limitation of this study was the relatively small sample size, which limited our ability to investigate other birth outcomes (i.e., PTB or IUGR) and evaluate interactions between air pollution exposure and potential effect modifiers such as maternal nutrition (Kannan et al. 2006). In addition, because we used daily mean levels of NO_2_ to temporally adjust the BTEX exposure model, identification of the second trimester as the most susceptible to BTEX exposure needs careful interpretation. Because NO_2_ and BTEX were highly correlated in space and time and both originate mainly from vehicle emissions in the study area, it remains unclear whether the more pronounced effect found for BTEX was independent of other traffic-related pollutants. Considering that NO_2_ is mainly a secondary pollutant (formed from the oxidation of NO primary emissions) and that LUR estimates of BTEX capture the influence of additional traffic emission sources such as parking lots, our results suggest that BTEX could be a more specific marker for exhaust toxins of concern for pregnancy in studies conducted within urban areas.

## Conclusions

We found an effect of exposure to traffic-related air pollutants (BTEX and, to a lesser extent, NO_2_) on birth weight among pregnant women who live in an urban area and spent < 2 hr/day in nonresidential outdoor locations. Although the magnitude of the association was higher for BTEX, the independent effect of different air pollutants with common emission sources remains to be determined. When possible, time–activity patterns during pregnancy should be considered to examine whether they may affect exposure misclassification. Overall, our findings add to a growing body of research linking intraurban variations of vehicle exhaust pollutants and reduced birth weight. Even being small, adverse reproductive effects of air pollution may have a considerable public health impact at the population level given the ubiquity of air pollution exposure (Slama et al. 2008a). This study reinforces the importance of developing strategies for air pollution prevention in a context of urban planning and management.

## Figures and Tables

**Table 1:** Characteristics of the study population (*n* = 570).

Variable	No. (%)	Birth weight (g)	*p*-Value^a^
Categorized variable			
Child’s sex			< 0.001
Male	288 (50.5)	3,316	
Female	282 (49.5)	3,177	
Season of conception			0.04
Spring	158 (27.7)	3,217	
Summer	167 (29.3)	3,248	
Fall	132 (23.2)	3,332	
Winter	113 (19.8)	3,188	
Tobacco smoking during pregnancy			< 0.001
0	467 (83.7)	3,277	
1–5 cigarettes/day	61 (10.9)	3,200	
> 5 cigarettes/day	30 (5.4)	3,023	
Passive smoking during pregnancy			0.03
Yes	241 (43.2)	3,207	
No	317 (56.8)	3,285	
Maternal parity			0.09
0	323 (56.9)	3,221	
≥ 1	245 (43.1)	3,283	
Maternal education			0.11
Primary education	166 (29.3)	3,197	
Secondary education	237 (41.8)	3,258	
University degree	164 (28.9)	3,274	
Maternal race/ethnicity			0.01
White/Caucasian	551 (96.8)	3,238	
Latin American	14 (2.5)	3,595	
Black	4 (0.7)	3,136	

Continuous variables	Median	IQR	*p*-Value^b^

Maternal age (years)	31.1	5.7	0.43
Gestational age (weeks)	39.9	1.9	< 0.001
Maternal height (cm)	162	8.3	0.01
Maternal prepregnancy weight (kg)	60	14	< 0.001
Paternal height (cm)	175	8	0.02
Paternal weight (kg)	79	15	0.01

a *p*-Values for comparing means by *t*-test or analysis of variance.

b *p*-Values of Pearson correlation coefficients between each continuous variable and birth weight.

**Table 2:** Distribution of 9-month and trimester exposures to NO_2_ and BTEX (μg/m^3^).

Pollutant (period)	Mean ± SD	Minimum	25th Percentile	Median	75th Percentile	Maximum
NO_2_						
9 months	32.17 ± 8.89	17.37	26.40	30.77	35.91	68.45
First trimester	32.66 ± 10.56	9.59	25.83	31.81	38.10	74.30
Second trimester	31.86 ± 10.57	10.33	24.98	30.97	36.98	77.47
Third trimester	32.67 ± 10.60	10.18	25.19	31.81	37.66	74.37
BTEX						
9 months	14.65 ± 5.52	3.95	10.25	14.65	18.67	27.63
First trimester	14.91 ± 6.21	2.44	9.78	14.68	19.57	29.51
Second trimester	14.49 ± 6.05	2.69	9.42	13.82	19.46	31.30
Third trimester	14.88 ± 6.24	2.62	9.86	14.03	19.58	31.69

**Table 3:** Spearman correlation coefficients between estimated air pollutant’s concentrations by 9-month and trimester exposures.

	NO_2_				BTEX			
Exposure	9 Months	First trimester	Second trimester	Third trimester	9 Months	First trimester	Second trimester	Third trimester
NO_2_								
9 months	1							
First trimester	0.79	1						
Second trimester	0.79	0.50	1					
Third trimester	0.80	0.45	0.46	1				
BTEX								
9 months	0.77	0.62	0.60	0.63	1			
First trimester	0.69	0.80	0.45	0.43	0.90	1		
Second trimester	0.71	0.48	0.80	0.44	0.90	0.74	1	
Third trimester	0.72	0.46	0.43	0.81	0.91	0.74	0.72	1

All correlation coefficients are significantly different from 0 (*p* < 0.01).

**Table 4:** Hours/day in specific activities/locations during pregnancy (reported in the 32nd week of pregnancy).

	Weekdays				Weekends			
Activity^a^	Mean ± SD	10th Percentile	Median	90th Percentile	Mean ± SD	10th Percentile	Median	90th Percentile
Indoor								
a. Home	15.1 ± 3.3	11.2	14.5	19.6	16.0 ± 3.1	12.0	16.0	20.0
b. Work	4.4 ± 3.8	0.0	5.0	9.0	0.2 ± 1.2	0.0	0.0	0.0
c. Other people’s houses	1.0 ± 1.4	0.0	0.5	3.0	2.3 ± 1.9	0.0	2.0	4.0
d. Other indoor environments	1.0 ± 0.8	0.0	1.0	2.0	1.6 ± 1.3	0.0	2.0	3.0
Outdoor								
e. Home	0.1 ± 0.4	0.0	0.0	0.0	0.2 ± 1.0	0.0	0.0	0.3
f. Work	0.2 ± 0.8	0.0	0.0	0.0	0.0 ± 0.5	0.0	0.0	0.0
g. Other outdoor environments	1.4 ± 1.1	0.3	1.0	3.0	2.7 ± 1.8	1.0	2.0	5.0
Walking^b^	0.9 ± 0.9	0.3	0.8	0.2	1.3 ± 1.1	0.5	1.0	2.0
Means of transportation^c^	0.8 ± 0.8	0.0	0.5	2.0	1.0 ± 0.7	0.0	1.0	2.0
Car	0.6 ± 0.8	0.0	0.5	1.5	0.9 ± 0.7	0.0	1.0	2.0
Bus	0.2 ± 0.9	0.0	0.0	0.5	0.0 ± 0.1	0.0	0.0	0.0
Metro/train	0.1 ± 0.3	0.0	0.0	0.0	0.0 ± 0.1	0.0	0.0	0.0
Total in nonresidential outdoor environments^d^								
(f + g)	1.6 ± 1.3	0.3	1.0	3.0	2.7 ± 1.8	1.0	2.0	5.0
Total at home								
(a + e)	15.1 ± 3.4	11.5	14.5	20.0	16.2 ± 3.0	12.5	16.4	20.0
Total in indoor environments								
(a + b + c + d)	21.5 ± 1.6	20.0	22.0	23.0	20.1 ± 2.2	17.0	20.5	22.5

See “Materials and Methods” for time–activity questions (a–h).

a Differences between weekdays and weekends are statistically significant for all the activities (Wilcoxon signed ranks test, *p* < 0.05).

b Women reported specifically the amount of time spent walking as part of the time spent in other outdoor environments.

c Mean, 10th percentile, median, and 90th percentile values for bicycle and motorcycle categories were 0.

d This activity refers to time spent in outdoor environments other than at the home address.

**Table 5:** Change (coefficient) in birth weight (g) for an IQR increase (μg/m^3^) in exposure to NO_2_ and BTEX at the entire pregnancy period and each trimester in 570 newborns from INMA-Sabadell.

	Birth weight [g (95% confidence interval)]		
	All women (*n* = 570)	Women who spent ≥ 15 hr/day at home (*n* = 276)	Women who spent < 2 hr/day in nonresidential outdoor environments (*n* = 259)
Crude			
NO_2_			
9 Months	−6.1 (−43.3 to 31.2)	−21.4 (−75.9 to 33.2)	−14.4 (−68.5 to 39.6)
First trimester	−4.5 (−45.2 to 36.2)	−6.1 (−67.4 to 55.2)	−1.2 (−61.4 to 59.1)
Second trimester	−18.1 (−57.5 to 21.4)	−45.2 (−103.4 to 12.9)	−46.2 (−103.7 to 11.2)
Third trimester	4.8 (−36.2 to 45.7)	−7.4 (−67.4 to 52.6)	6.7 (−52.2 to 65.6)
BTEX			
9 Months	−9.6 (−62.8 to 43.6)	−37.2 (−114.5 to 40.1)	−61.9 (−140.0 to 17.6)
First trimester	−10.9 (−66.1 to 44.3)	−27.3 (−110.3 to 55.7)	−50.0 (−132.0 to 32.1)
Second trimester	−26.4 (−84.5 to 31.7)	−68.0 (−152.8 to 16.8)	−98.5 (−184.0 to −13.0)*
Third trimester	6.5 (−47.7 to 60.8)	−15.8 (−94.7 to 63.0)	−29.7 (−110.7 to 51.2)

Adjusted (each trimester separately)^a^			
NO_2_			
9 Months	8.8 (−23.8 to 41.5)	8.6 (−37.9 to 55.2)	−18.6 (−66.3 to 29.1)
First trimester	3.3 (−33.2 to 39.7)	9.8 (−43.2 to 62.9)	4.2 (−49.3 to 57.6)
Second trimester	3.7 (−31.1 to 38.4)	−1.5 (−50.9 to 47.9)	−42.3 (−92.1 to 7.4)
Third trimester	16.8 (−18.8 to 52.4)	15.8 (−35.1 to 66.6)	−9.0 (−60.5 to 42.6)
BTEX			
9 Months	−7.6 (−54.9 to 39.8)	−13.0 (−79.3 to 53.3)	−76.6 (−146.3 to −7.0)*
First trimester	−12.0 (−62.0 to 38.0)	−16.3 (−87.8 to 55.2)	−52.5 (−125.8 to 20.8)
Second trimester	−13.3 (−65.1 to 38.4)	−22.5 (−95.1 to 50.0)	−101.9 (−176.2 to −27.6)*
Third trimester	2.5 (−45.3 to 50.4)	−1.4 (−68.8 to 66.0)	−59.7 (−130.9 to 11.5)

Adjusted (all trimesters together)^b^			
NO_2_			
First trimester	−7.6 (−56.8 to 41.5)	12.7 (−63.0 to 88.4)	54.8 (−18.5 to 128.0)
Second trimester	−5.8 (−52.7 to 41.0)	−22.8 (−90.7 to 45.0)	−74.7 (−140.4 to −9.0)*
Third trimester	24.6 (−22.1 to 71.3)	22.1 (−46.1 to 90.2)	0.9 (−64.4 to 66.3)
BTEX			
First trimester	−19.2 (−101.0 to 62.6)	−13.4 (−139.9 to 113.0)	60.8 (−61.8 to 183.5)
Second trimester	−21.7 (−104.6 to 61.3)	−42.3 (−161.4 to 77.0)	−137.3 (−252.6 to −22.0)*
Third trimester	30.0 (−44.8 to 104.9)	35.8 (−74.8 to 146.4)	−16.7 (−123.3 to 89.8)

a Adjusted for child’s sex, gestational age, season of conception, parity, maternal educational level, maternal smoking during pregnancy, maternal height and prepregnancy weight, and paternal height.

b Adjusted for above variables and exposures to the same pollutant during the other two trimesters.

* *p* < 0.05.

## References

[b1-ehp-117-1322] Agency for Toxic Substances and Disease Registry (2004). Interaction Profile for Benzene, Toluene, Ethylbenzene, and Xylenes (BTEX).

[b2-ehp-117-1322] Aguilera I, Sunyer J, Fernández-Patier R, Hoek G, Aguirre-Alfaro A, Meliefste K (2008). Estimation of outdoor NO_x_, NO_2_, and BTEX exposure in a cohort of pregnant women using land use regression modeling. Environ Sci Technol.

[b3-ehp-117-1322] Alexopoulos EC, Chatzis C, Linos A (2006). An analysis of factors that influence personal exposure to toluene and xylene in residents of Athens, Greece. BMC Public Health.

[b4-ehp-117-1322] Bell ML, Ebisu K, Belanger K (2007). Ambient air pollution and low birth weight in Connecticut and Massachusetts. Environ Health Perspect.

[b5-ehp-117-1322] Blair EM, Liu Y, de Klerk NH, Lawrence DM (2005). Optimal fetal growth for the Caucasian singleton and assessment of appropriateness of fetal growth: an analysis of a total population perinatal database. BMC Pediatr.

[b6-ehp-117-1322] Brauer M, Lencar C, Tamburic L, Koehoorn M, Demers P, Karr C (2008). A cohort study of traffic-related air pollution impacts on birth outcomes. Environ Health Perspect.

[b7-ehp-117-1322] European Commission (1999). Council Directive 1999/30/EC of 22 April 1999 Relating to Limit Values for Sulphur Dioxide, Nitrogen Dioxide and Oxides of Nitrogen, Particulate Matter and Lead in Ambient Air. http://eur-lex.europa.eu/LexUriServ/LexUriServ.do?uri=OJ:L:1999:163:0041:0060:EN:PDF.

[b8-ehp-117-1322] Fell DB, Dodds L, King WD (2004). Residential mobility during pregnancy. Paediatr Perinat Epidemiol.

[b9-ehp-117-1322] Forastiere F, Stafoggia M, Tasco C, Picciotto S, Agabiti N, Cesaroni G (2007). Socioeconomic status, particulate air pollution, and daily mortality: differential exposure or differential susceptibility. Am J Ind Med.

[b10-ehp-117-1322] Gilliland F, Avol E, Kinney P, Jerrett M, Dvonch T, Lurmann F (2005). Air pollution exposure assessment for epidemiologic studies of pregnant women and children: lessons learned from the Centers for Children’s Environmental Health and Disease Prevention Research. Environ Health Perspect.

[b11-ehp-117-1322] Glinianaia SV, Rankin J, Bell R, Pless-Mulloli T, Howel D (2004). Particulate air pollution and fetal health: a systematic review of the epidemiologic evidence. Epidemiology.

[b12-ehp-117-1322] Gouveia N, Bremner SA, Novaes HM (2004). Association between ambient air pollution and birth weight in São Paulo, Brazil. J Epidemiol Community Health.

[b13-ehp-117-1322] Ha EH, Hong YC, Lee BE, Woo BH, Schwartz J, Christiani DC (2001). Is air pollution a risk factor for low birth weight in Seoul?. Epidemiology.

[b14-ehp-117-1322] Hansen C, Neller A, Williams G, Simpson R (2007). Low levels of ambient air pollution during pregnancy and fetal growth among term neonates in Brisbane, Australia. Environ Res.

[b15-ehp-117-1322] Hazenkamp-von Arx ME, Götschi T, Ackermann-Liebrich U, Bono R, Burney P, Cyrys J (2004). PM_2.5_ and NO_2_ assessment in 21 European study centres of ECRHS II: annual means and seasonal differences. Atmos Environ.

[b16-ehp-117-1322] Hoek G, Brunekreef B, Goldbohm S, Fischer P, van den Brandt PA (2002). Association between mortality and indicators of traffic-related air pollution in the Netherlands: a cohort study. Lancet.

[b17-ehp-117-1322] Jedrychowski W, Bendkowska I, Flak E, Penar A, Jacek R, Kaim I (2004). Estimated risk for altered fetal growth resulting from exposure to fine particles during pregnancy: an epidemiologic prospective cohort study in Poland. Environ Health Perspect.

[b18-ehp-117-1322] Kannan S, Misra DP, Dvonch JT, Krishnakumar A (2006). Exposures to airborne particulate matter and adverse perinatal outcomes: a biologically plausible mechanistic framework for exploring potential effect modification by nutrition. Environ Health Perspect.

[b19-ehp-117-1322] Lacasaña M, Esplugues A, Ballester F (2005). Exposure to ambient air pollution and prenatal and early childhood health effects. Eur J Epidemiol.

[b20-ehp-117-1322] Lebret E, Briggs D, Van Reeuwijk H, Fischer P, Smallbone K, Harssema H (2000). Small area variations in ambient NO_2_ concentrations in four European areas. Atmos Environ.

[b21-ehp-117-1322] Lee BE, Ha EH, Park HS, Kim YJ, Hong YC, Kim H (2003). Exposure to air pollution during different gestational phases contributes to risks of low birth weight. Hum Reprod.

[b22-ehp-117-1322] Liu S, Krewski D, Shi Y, Chen Y, Burnett RT (2003). Association between gaseous ambient air pollutants and adverse pregnancy outcomes in Vancouver, Canada. Environ Health Perspect.

[b23-ehp-117-1322] Liu S, Krewski D, Shi Y, Chen Y, Burnett RT (2007). Association between maternal exposure to ambient air pollutants during pregnancy and fetal growth restriction. J Expo Sci Environ Epidemiol.

[b24-ehp-117-1322] Mannes T, Jalaludin B, Morgan G, Lincoln D, Sheppeard V, Corbett S (2005). Impact of ambient air pollution on birth weight in Sydney, Australia. Occup Environ Med.

[b25-ehp-117-1322] Muñoz F (2003). Lock living: urban sprawl in Mediterranean cities. Cities.

[b26-ehp-117-1322] Murray LJ, O’Reilly DP, Betts N, Patterson CC, Davey SG, Evans AE (2000). Season and outdoor ambient temperature: effects on birth weight. Obstet Gynecol.

[b27-ehp-117-1322] Nethery E, Brauer M, Janssen P (2009). Time-activity patterns of pregnant women and changes during the course of pregnancy. J Expo Sci Environ Epidemiol.

[b28-ehp-117-1322] Nethery E, Leckie SE, Teschke K, Brauer M (2008). From measures to models: an evaluation of air pollution exposure assessment for epidemiologic studies of pregnant women. Occup Environ Med.

[b29-ehp-117-1322] O’Neill MS, Jerrett M, Kawachi I, Levy JI, Cohen AJ, Gouveia N (2003). Health, wealth, and air pollution: advancing theory and methods. Environ Health Perspect.

[b30-ehp-117-1322] Ribas-Fitó N, Ramón R, Ballester F, Grimalt J, Marco A, Olea N (2006). Child health and the environment: the INMA Spanish Study. Paediatr Perinat Epidemiol.

[b31-ehp-117-1322] Ritz B, Wilhelm M (2008). Ambient air pollution and adverse birth outcomes: methodologic issues in an emerging field. Basic Clin Pharmacol Toxicol.

[b32-ehp-117-1322] Rivas-Lara I (2008). Variabilitat temporal i geogràfica i caracterització química de la contaminació atmosfèrica particulada a Sabadell [in Catalan] [MSc thesis].

[b33-ehp-117-1322] Salam MT, Millstein J, Li YF, Lurmann FW, Margolis HG, Gilliland FD (2005). Birth outcomes and prenatal exposure to ozone, carbon monoxide, and particulate matter: results from the Children’s Health Study. Environ Health Perspect.

[b34-ehp-117-1322] Sinclair KD, Lea RG, Rees WD, Young LE (2007). The developmental origins of health and disease: current theories and epigenetic mechanisms. Soc Reprod Fertil.

[b35-ehp-117-1322] Slama R, Darrow L, Parker J, Woodruff TJ, Strickland M, Nieuwenhuijsen M (2008a). Meeting report: atmospheric pollution and human reproduction. Environ Health Perspect.

[b36-ehp-117-1322] Slama R, Khoshnood B, Kaminski M (2008b). How to control for gestational age in studies involving environmental effects on fetal growth [Letter]. Environ Health Perspect.

[b37-ehp-117-1322] Slama R, Morgenstern V, Cyrys J, Zutavern A, Herbarth O, Wichmann HE (2007). Traffic-related atmospheric pollutants levels during pregnancy and offspring’s term birth weight: a study relying on a land-use regression exposure model. Environ Health Perspect.

[b38-ehp-117-1322] Šrám RJ, Binková B, Dejmek J, Bobak M (2005). Ambient air pollution and pregnancy outcomes: a review of the literature. Environ Health Perspect.

[b39-ehp-117-1322] Tustin K, Gross J, Hayne H (2004). Maternal exposure to first-trimester sunshine is associated with increased birth weight in human infants. Dev Psychobiol.

[b40-ehp-117-1322] Wang L, Pinkerton KE (2007). Air pollutant effects on fetal and early postnatal development. Birth Defects Res C Embryo Today.

[b41-ehp-117-1322] Westerway SC, Davison A, Cowell S (2000). Ultrasonic fetal measurements: new Australian standards for the new millennium. Aust N Z J Obstet Gynaecol.

[b42-ehp-117-1322] Wilhelm M, Ritz B (2005). Local variations in CO and particulate air pollution and adverse birth outcomes in Los Angeles County, California, USA. Environ Health Perspect.

